# Predictors of Massive Transfusion in Patients With Hollow Organ Injury After Blunt Multiple Trauma: A Cohort of Blunt Bowel Mesenteric Injury

**DOI:** 10.1155/emmi/5286788

**Published:** 2026-04-13

**Authors:** Ting-Min Hsieh, Po-Chun Chuang, Chun-Ting Liu, Bei-Yu Wu, Ching-Hua Hsieh, Fu-Jen Cheng

**Affiliations:** ^1^ Division of Trauma, Department of Surgery, Kaohsiung Chang Gung Memorial Hospital and Chang Gung University College of Medicine, 123 Ta Pei Road Niao-Song District, Kaohsiung, Taiwan, cgmh.org.tw; ^2^ Department of Emergency, Kaohsiung Chang Gung Memorial Hospital and Chang Gung University College of Medicine, 123 Ta Pei Road Niao-Song District, Kaohsiung, Taiwan, cgmh.org.tw; ^3^ Department of Chinese Medicine, Kaohsiung Chang Gung Memorial Hospital and Chang Gung University College of Medicine, 123 Ta Pei Road Niao-Song District, Kaohsiung, Taiwan, cgmh.org.tw; ^4^ Division of Plastic Surgery, Department of Surgery, Kaohsiung Chang Gung Memorial Hospital and Chang Gung University College of Medicine, 123 Ta Pei Road Niao-Song District, Kaohsiung, Taiwan, cgmh.org.tw; ^5^ Institute for Translational Research in Biomedicine, Kaohsiung Chang Gung Memorial Hospital, Kaohsiung, Taiwan, cgmh.org.tw

**Keywords:** BAT, blunt abdominal trauma, blunt bowel mesenteric injuries, laparotomy, massive transfusion

## Abstract

**Background:**

Massive transfusion (MT) is life‐saving for patients with exsanguination, especially after blunt abdominopelvic trauma, due to subtle manifestations. Blunt bowel mesenteric injuries (BBMIs), besides their potential risk of peritonitis, are still one of the few indications for emergency laparotomy for hemorrhagic shock in the era of nonoperative management. Early prediction of the need for MT defined as using ≥ 10 units of packed red blood cells (PRBCs) in 24 h and activation of the MT protocol (MTP) is a critical aspect in resuscitation. Current scoring systems predicting MT are usually laboratory data or hemodynamic status–dependent, which are limited by the time‐consuming and dynamic characteristics of trauma; thus, they seem to lack objectivity in patients with BBMI due to dramatic clinical deterioration. The present study aimed to determine the predictors of associated injuries contributing to the requirement for MT in patients who underwent surgical BBMI.

**Methods:**

This retrospective study reviewed the data of hospitalized patients with trauma between 2009 and 2022. The patients were divided based on the presence or absence of MT before emergency laparotomy. Associated injury parameters and bowel mesenteric injury characteristics were used in multivariate regression analysis to identify independent predictors of MT.

**Results:**

A total of 163 adult patients with surgically proven BBMI were enrolled in the study. The overall patients with MT were 30.6% (50/163). Compared to the MT (−) group, BBMI patients receiving MT had worse clinical injury severity, vital signs, and prognosis; patients receiving MT had significant a lower initial hemoglobin level and higher percentages of receiving PRBC (11.15 mg/dL vs. 13.10 mg/dL, *p* < 0.001 and 47% vs. 42%, *p* < 0.001) and required more volume of PRBC at emergency department (ED) (5.5 units vs. 0 units, *p* < 0.001) as compared to the MT (−) group. Besides, patients with MT administered more amounts of PRBC within 24 h and at operation room in comparison with patients without MT (16 units vs. 2 units, *p* < 0.001 and 8 units vs. 0 units, *p* < 0.001). Patients with MT involved with more isolated mesenteric injury or combined injury and had both higher complications and overall mortality rates (94% vs. 55.8%, *p* < 0.001 and 32% vs. 4.4%, *p* < 0.001). In multivariate analysis, the presence of traumatic brain injury (TBI) (odds ratio [OR] = 6.7, 95% confidence intervals [CIs]: 1.66–27.02) and pelvic fracture (OR = 6.01, 95% CIs = 1.45–24.89) was identified as an independent predictor of MT after adjusting for confounding factors.

**Conclusions:**

For patients with BBMI, one‐third require MT prior to laparotomy, necessitating early activation of the MTP. When BBMI patients present with hemodynamic instability or higher injury severity, particularly in the presence of concomitant TBI or pelvic fractures, trauma surgeons should initiate more aggressive resuscitation to prevent therapeutic delays.

## 1. Introduction

Hemorrhage is the chief cause and a potential preventable mortality among trauma patients, as the odds of mortality decrease by 5% every minute from early activation of the massive transfusion protocol (MTP) to blood product administration [1]. Numerous scoring systems and protocols have been established to predict the need for MT in trauma victims [2–5]. Among these variables in different scoring systems, it is not difficult to find that they have many similarities with the involvement of patients’ vital signs, laboratory data, results of focused assessment with sonography in trauma (FAST), and the occurrence of pelvic fracture [2, 3, 5, 6]. Abdominal trauma appears to be common in patients with severe exsanguination.

Blunt abdominal trauma (BAT) is an entity that can cause significant morbidity and mortality at the emergency department (ED) with easy neglect, particularly in the patients of poor consciousness or multitraumatized status. Although the liver and spleen are the most vulnerable organs in patients after BAT, the stress associated with catastrophic bleeding in trauma surgeons has decreased due to the liberal use of angioembolization in the era of nonoperative management (NOM). In contrast, although blunt bowel mesenteric injury (BBMI) is the third most common injury, with rareness but potential sepsis [6], it is another often negligible cause of exsanguination and still requires less laparotomy after BAT [7]. Given the low incidence, subtle clinical signs, noneasily interpreted imaging studies, and high necessity of emergency laparotomy, it occasionally leads to delayed diagnosis and increased morbidity and mortality [6, 7]. To decrease the missed diagnosis of significant BBMI, some prediction scores based on the computed tomography (CT) image, laboratory data, and clinical examination were developed and validated retrospectively [8].

Nevertheless, in light of the dramatically and subtly deteriorated course, it is not practical to predict MT clinically for the most existing scoring systems due to the availability of the time‐consuming laboratory data, or the initial vital signs, although they are all helpful adjuvant tools in clinical decision‐making [2–5]. It was found that the shock index (SI) has been used to predict MT in patients with liver and spleen injuries [9]; however, studies related to MT in patients with BBMI are scarce, particularly in patients with surgical BBMI.

The study by El‐Menyar et al. [4] used FAST, SI, and serum lactate to develop a novel FASILA score (0–6) and reported that patients with abdominal trauma with a full FASILA score of 6 had a possibility of 68% needing MT and 85% needing exploratory laparotomy. Although the aforementioned scoring systems are easily used and validated, their prediction of MT is mainly based on the shock level from the patient’s laboratory data or hemodynamic status. In addition, the vital signs, laboratory data, and FAST results were all clinically dynamic after trauma. Therefore, there seems to be a lack of objectivity in using these scoring systems to predict MT or requirement for laparotomy in poly‐trauma patients with concomitant BBMI. Moreover, in contrast to solid organ injuries, significant BBMI is more easily overlooked because NOM is not evidence‐based; however, BBMI is only excluded with laparotomy that is not without risk [10].

The presence of significant BBMI often combined with multiple trauma, which may exacerbate the hemodynamic instability, interrupts the decision‐making and complicated the treatment course in trauma bay [11]. Although the wide use of CT scan, highly validated performance of prediction scores [8], and growing publication [10, 11] had made trauma surgeon more vigilant with the entity, the association of MT between its associated injury and BBMI is less discussed. In contrast to previous scoring tools applying to solid organ injuries or requiring dynamic data, we aimed to identify a simple, expeditious, and nondynamic method to predict the MT in multitraumatized patients with concomitant BBMI and prompt trauma surgeon to treat more actively. This study aimed to explore the association between MT and surgical BBMI in terms of the associated injuries.

## 2. Methods

### 2.1. Ethics Statement

This study was approved by the Institutional Review Board (IRB) of Chang Gung Memorial Hospital (Approval number: 202500868B0). The need for informed consent was waived according to IRB regulations.

### 2.2. Study Population

**FIGURE 1:**
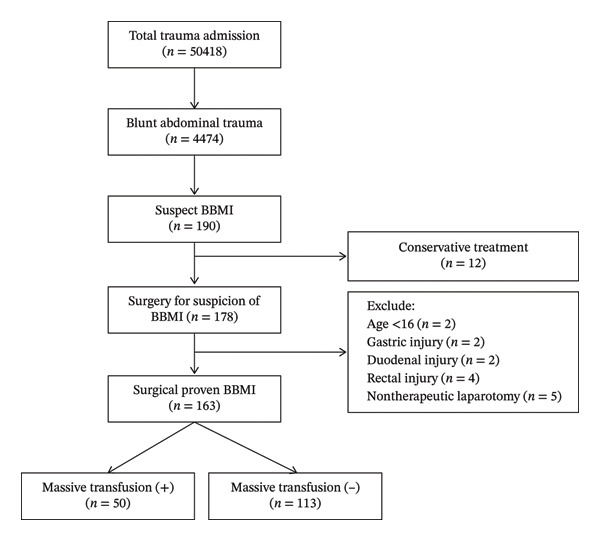
Enrollment flowchart for patients with surgical blunt bowel mesenteric injury.

This retrospective study reviewed all data added to the Trauma Registry System from January 1, 2009, to December 31, 2022, in a 2686‐bed facility and Level I regional trauma center that provides care to trauma patients in southern Taiwan. All data were prospectively collected from the medical records of hospitalized patients with trauma and retrospectively analyzed. The patients enrolled in this study were adult trauma patients (> 16 years of age) who underwent emergency therapeutic laparotomy, which is defined as the use of procedures to repair or resect the bowel/mesentery or to control active bleeding for suspected BBMI. During the 14‐year investigation period, only patients with surgically proven BBMI were included. Patients with isolated stomach, duodenal, or rectal injuries were excluded. Finally, the included patients were categorized into two groups on the basis of the presence of receiving MT, commonly defined as 10 or more units of packed red blood cells (PRBCs) within the first 24 h of admission [12], during the period between arrival at the initial ED and emergency laparotomy. The patient selection process for the study cohort is illustrated in Figure 1. Patients who received MT before laparotomy were compared with those who did not receive MT, and associated injuries were assessed for their ability to predict the need for MT.

### 2.3. Study Parameters

The following variables were extracted for each patient: demographic data such as age, sex, and body mass index (BMI); clinical and trauma data such as injury severity score (ISS), new ISS (NISS), trauma resuscitation ISS (TRISS), revised trauma score (RTS), and abbreviated injury score (AIS) over the head, face, chest, abdomen, and extremities; vital signs at the ED, including systolic blood pressure (SBP), heart rate, respiratory rate, and Glasgow Coma Scale (GCS) score; injury mechanisms; clinical presentation such as the hemoglobin level upon arrival at the ED, incidence of intubation, and tube thoracostomy at the ED; status of chock (defined as SBP ≦ 90 Hg at the ED); status of blood transfusion (BT), including the incidence of BT at the ED; amount of PRBCs and fresh frozen plasma (FFP) transfused at the ED within 24 h and at the operating room and ward; and operative findings, including the incidence of isolated small bowel injury (defined as small bowel injury with AIS abdomen ≥ 2, including ischemia, rupture, serosal injury, or hematoma), isolated colon injury (defined as colon injury with AIS abdomen ≥ 2, including ischemia, rupture, serosal injury, or hematoma), isolated mesentery injury (defined as mesenteric injury with AIS abdomen ≥ 2), or combined injury (defined as either small bowel or colon injury concomitant with mesenteric injury, including ischemia, rupture, serosa injury, or hematoma), and operative blood loss and delayed operation (defined as patients whose emergency laparotomy was performed during admission to the intensive care unit [ICU] or ward under a miss diagnosis rather than initially at the ED). Outcome data: Morbidity and mortality were classified according to cause as either bowel injury or bleeding injury, 24‐h mortality, or length of stay (LOS) in the hospital and ICU. Morbidities were identified during chart reviews based on standard definitions. Bowel‐related mortality was defined as mortality due to abdominal sepsis following surgery. Exsanguination‐related mortality was defined as mortality due to surgically proven hemorrhagic shock resulting from bowel or mesenteric bleeding.

### 2.4. Statistical Analysis

Data were analyzed using IBM SPSS Statistics for Windows (Version 20.0; IBM Corp., Armonk, NY, USA). Continuous variables are reported as medians and interquartile ranges. Considering the potential impact of the small sample size on our statistical analysis, we treated the data as non‐normally distributed. Therefore, the Mann–Whitney U test was used to analyze these continuous variables. In the initial phase, factors such as age, sex, BMI, associated injuries, and BBMI injury patterns based on intraoperative findings were identified as significant in the univariate analysis. These variables were subsequently incorporated into a binary regression model to identify the independent predictors of groups requiring MT, allowing for the adjustment for potential confounders. Multicollinearity among the candidate predictors was assessed using variance inflation factors (VIFs). All variables included in the regression model had VIF values < 3, indicating a low level of multicollinearity and acceptable independence among predictors (supporting Table S1). Sensitivity analyses were performed by excluding early deaths (within 24 h after ED visit) and by stratifying the cohort according to study period (supporting Table S2 and S3). In addition, a reduced regression model including only TBI and pelvic fracture was constructed to evaluate the robustness of the findings (supporting Table S4). Because several variables showed sparse data and wide confidence intervals (CIs) in the conventional logistic regression model, we performed an additional analysis using Firth penalized logistic regression to reduce potential small‐sample bias and address possible separation issues. The Firth method has been recommended for logistic regression analyses when the number of events is limited or when sparse data may lead to unstable estimates. To analyze the temporal relationship between MT and morbidity, Kaplan–Meier analysis was used, and the log‐rank test was applied to compare the morbidity and mortality curves between the MT(+) and MT(−) groups. The threshold for statistical significance was set at *p* < 0.05. Odds ratios (ORs) with corresponding 95% CIs and *p* values were calculated. All statistical analyses were primarily performed using IBM SPSS Statistics (Version 25; IBM Corp., Armonk, NY, USA). Because SPSS does not provide Firth penalized logistic regression, the Firth regression analysis and Kaplan–Meier survival analysis were performed using R software (R Foundation for Statistical Computing, Vienna, Austria). A two‐sided *p* value < 0.05 was considered statistically significant.

## 3. Results

### 3.1. Patient Groups, Characteristics, Clinical Presentation, and Outcomes

The patient selection process is shown in Figure 1. During the 14‐year study period, 50,418 trauma patients were admitted to our Level I trauma center. After excluding patients with isolated gastric, duodenal, or rectal injuries, 163 adult patients who underwent therapeutic laparotomy for BBMI were included in the final analysis. Of these patients with surgically proven BBMI, 50 (30.6%) received MTs and were defined as the MT (+) group. The patients in the two groups were similar in terms of age, sex, and injury mechanisms. When compared to the MT(−) group, the MT(+) group had more severe anatomic injuries due to ISS (25 vs. 9, *p* < 0.001) and NISS (27 vs. 16, *p* < 0.001), physiological injuries due to RTS (6.90 vs. 7.84, *p* < 0.001), worse ED vital signs, more critical ED clinical presentation, and a greater percentage or amount of transfusion at the ED. Regarding the intraoperative findings, the MT(−) group had a significantly higher incidence of isolated bowel injury and isolated colon injury (*p* < 0.001) than the MT(+) group, whereas the MT(+) group had a significantly higher incidence of isolated mesenteric injury or combined injury (*p* < 0.001) and a significantly greater amount of operative blood loss than the MT(−) group (*p* < 0.001). The two groups had a similar frequency of delayed surgeries (14.2% vs. 4%, *p* = 0.056). Additionally, the overall morbidity and mortality rates were greater in the MT(+) group than in the MT(−) group (94% vs. 55.8% [*p* < 0.001] and 32% vs. 4.4% [*p* < 0.001], respectively) (Table 1). On the basis of the results of the Kaplan–Meier analysis, patients in the MT(+) group had a higher and earlier mortality rate than those in the MT(−) group (*p* < 0.001) (Figure 1).

**TABLE 1:** Clinical and injury characteristics of patients with BBMI according to the massive transfusion group.

	Overall (*N* = 163)	MT (−) (*n* = 113)	MT (+) (*n* = 50)	*p* value
Age	47 (31–60)	49 (28–61)	44 (36–55)	0.776
Male sex	132 (81%)	91 (80.5%)	41 (82%)	0.826
Body mass index	24.6 (21.5–27.5)	24.1 (21.1–26.8)	25.95 (22.8–28.4)	0.010
ISS	16 (9–25)	10 (9–18)	25 (16–32)	< 0.001
ISS ≥ 16 (%)	87 (53.4)	47 (41.6)	40 (80)	< 0.001
ISS ≥ 25 (%)	41 (25.2)	12 (10.6)	29 (58)	< 0.001
NISS	18 (9–27)	16 (9–22)	27 (18–38)	< 0.001
TRISS	0.99 (0.94–0.67)	0.98 (0.96–0.99)	0.96 (0.99–0.94)	< 0.001
RTS	7.84 (7.108–7.84)	7.84 (7.84–7.84)	6.904 (6–7.84)	< 0.001
ED vital sign				
SBP (mm/Hg)	116 (91–133)	119 (101–137)	90.5 (73–122)	< 0.001
HR (/min)	96 (80–118)	91 (79–109)	105 (83–130)	0.024
RR (/min)	20 (18–20)	20 (18–20)	20 (16–22)	0.675
GCS	15 (14–15)	15 (15–15)	12 (5–15)	< 0.001
Mechanism				
Motorcycle (%)	82 (50.3)	56 (49.6)	26 (52)	0.515
Car (%)	42 (25.8)	28 (24.8)	14 (28)	
Fall (%)	5 (3.1)	5 (4.4)	0 (0)	
High fall (%)	6 (3.7)	5 (4.4)	1 (2)	
Pedestrian (%)	8 (4.9)	5 (4.4)	3 (6)	
Assault (%)	5 (3.1)	4 (3.5)	1 (2)	
Bicycle (%)	7 (4.3)	5 (4.4)	2 (4)	
Impact (%)	8 (4.9)	5 (4.4)	3 (6)	
Clinical presentation				
ED hemoglobin, mg/dL	12.6 (10.7–14.2)	13.1 (11.6–14.4)	11.15 (8.9–12.9)	< 0.001
ED intubation (%)	36 (22.1)	9 (8)	27 (54)	< 0.001
ED chest tube (%)	31 (19)	15 (13.3)	16 (32)	0.005
Shock	74 (45.4)	27 (23.9)	47 (94.0)	< 0.001
Blood transfusion				
B/T at ED (%)	89 (54.6)	42 (37.2)	47 (94)	< 0.001
ED pack RBC (U)	2 (0–4)	0 (0–2)	5.5 (2–8)	< 0.001
ED FFP (U)	0 (0–2)	0 (0–0)	3.5 (0–6)	< 0.001
24‐h pack RBC (U)	4 (0–12)	2 (0–4)	16 (12–24)	< 0.001
24‐h FFP (U)	2 (0–8)	0 (0–4)	12 (8–20)	< 0.001
OR pack RBC (U)	2 (0–6)	0 (0–2)	8 (6–12)	< 0.001
OR FFP (U)	0 (0–4)	0 (0–2)	6 (4–12)	< 0.001
Ward pack RBC (U)	0 (0–4)	0 (0–2)	2 (0–10)	< 0.001
Ward FFP (U)	0 (0–4)	0 (0–2)	3 (0–12)	< 0.001
Operative finding:				
Isolated bowel injury (%)	42 (25.8)	41 (36.3)	1 (2)	< 0.001
Isolated colon injury (%)	18 (11)	17 (15)	1 (2)	
Isolated mesentery injury (%)	51 (31.3)	27 (23.9)	24 (48)	
Combined injury (%)	52 (31.9)	28 (24.8)	24 (48)	
Operation blood loss (ml)	500 (100–2000)	200 (50–900)	3000 (1500–5000)	< 0.001
Delayed operation (%)	18 (11)	16 (14.2)	2 (4)	0.056
Outcome				
Morbidity (%)	110 (67.5)	63 (55.8)	47 (94)	< 0.001
Mortality (%)	21 (12.9)	5 (4.4)	16 (32)	< 0.001
24‐h mortality (%)	7 (4.3)	3 (2.7)	4 (8)	0.203
Bowel‐related mortality (%)	5 (3.1)	2 (1.8)	3 (6)	0.169
Exsanguination mortality (%)	11 (6.7)	2 (1.8)	9 (18)	< 0.001
ICU length of stay (day)	3 (2–7)	2 (1–5)	4 (2–16)	< 0.001
Hospitalization LOS (day)	17 (10–31)	16 (11–27)	20.5 (8–44)	0.953

*Note:* Data were presented as a number (percentage) and median IQR (25%–75%).

### 3.2. Predicting Factors of MT

Univariate and subsequent multivariate analyses identified the injury‐related risk factors associated with MT (Table 2). Univariate analyses revealed that BMI (*p* = 0.01), vessel injury (*p* = 0.005), traumatic brain injury (TBI) (*p* < 0.001), rib fracture (*p* = 0.001), and pelvic fracture (*p* = 0.001) were associated with an increased risk of MT. Multivariate analyses revealed that TBI (OR = 6.7, 95% CI] = 1.66–27.02) and pelvic fracture (OR = 6.01, 95% CI = 1.45–24.89) were independently associated with the presence of MT. Furthermore, it is expected that isolated mesentery injury (OR = 23.11, 95% CI = 2.51–212.66) and combined injury (OR = 27.23, 95% CI = 2.96–250.79) are strongly independent predictors of MT. Additional analysis using Firth penalized logistic regression yielded results consistent with the primary multivariable logistic regression model. TBI, pelvic fracture, isolated mesenteric injury, and combined injury remained significant predictors of MT. The detailed results are shown in Supporting Table S5.

**TABLE 2:** Predictors for massive transfusion.

	Univariate analysis		Multivariate analysis	
	OR (95% CI)	*p* value	AOR (95% CI)	*p* value
Age	0.99 (0.98–1.02)	0.855	0.99 (0.97–1.02)	0.792
Male sex	1.10 (0.47–2.59)	0.826	1.82 (0.57–5.85)	0.314
Body mass index	1.12 (1.03–1.23)	0.010	1.07 (0.94–1.23)	0.302
Vessel	3.62 (1.46–8.95)	0.005	1.57 (0.53–4.62)	0.417
Traumatic brain injury	7.59 (2.53–22.73)	< 0.001	6.70 (1.66–27.02)	0.008
Lung contusion	2.26 (0.86–5.97)	0.099	0.92 (0.23–3.72)	0.912
Rib fracture	3.96 (1.70–9.21)	0.001	2.29 (0.57–9.12)	0.242
Hemopneumothorax	1.71 (0.73–4.01)	0.218	0.64 (0.13–3.16)	0.586
Pelvic fracture	5.32 (1.97–14.35)	0.001	6.01 (1.45–24.89)	0.013
Low limb fracture	1.78 (0.78–4.09)	0.171	1.11 (0.35–3.55)	0.860
Operation finding (compare to isolated bowel injury)				
Isolated colon injury	2.41 (0.14–40.82)	0.542	0.85 (0.038–19.45)	0.916
Isolated mesentery injury	36.44 (4.65–285.52)	0.001	23.11 (2.51–212.66)	0.006
Combined injury	35.14 (4.49–274.98)	0.001	27.23 (2.96–250.79)	0.004

Abbreviations: AOR, adjusted odds ratio; OR, odds ratio.

### 3.3. Injury Severity and Injury Pattern

The distribution of AIS injuries in each body region in the two groups is shown in Table 3. The MT(+) group had significantly higher injury severity over AIS of the head (*p* < 0.001), chest (*p* = 0.05), abdomen (*p* < 0.001), and extremities (*p* = 0.014) than the MT(−) group. Furthermore, the MT(+) group had more frequency in AIS head ≥ 2 (*p* < 0.001), AIS head ≥ 3 (*p* < 0.001), AIS chest ≥ 3 (*p* = 0.034), and AIS extremities ≥ 2 (*p* = 0.006) compared with the MT(−) group.

**TABLE 3:** Severity of injury in body regions of patients with BBMI according to the massive transfusion.

	Overall (*N* = 163)	MT (−) (*n* = 113)	MT (+) (*n* = 50)	*p* value
AIS head	0 (0–0)	0 (0–0)	0 (0–2)	< 0.001
AIS face	0 (0–0)	0 (0–0)	0 (0–0)	0.413
AIS chest	0 (0–1)	0 (0–0)	0 (0–3)	0.05
AIS abdomen	3 (3–4)	3 (3–3)	3.5 (3–4)	< 0.001
AIS extremities	0 (0–2)	0 (0–2)	0 (0–2)	0.014
AIS head ≥ 2 (%)	21 (12.9)	7 (6.2)	14 (28)	< 0.001
AIS head ≥ 3 (%)	15 (9.2)	3 (2.7)	12 (24)	< 0.001
AIS face ≥ 2 (%)	11 (6.7)	6 (5.3)	5 (10)	0.340
AIS face ≥ 3 (%)	0 (0)	0 (0)	0 (0)	
AIS chest ≥ 2 (%)	39 (23.9)	21 (18.6)	18 (36)	0.119
AIS chest ≥ 3 (%)	35 (21.5)	18 (15.9)	17 (34)	0.034
AIS abdomen ≥ 2 (%)	163 (100)	113 (100)	50 (100)	
AIS abdomen ≥ 3 (%)	148 (90.8)	100 (88.5)	48 (96)	0.498
AIS extremities ≥ 2 (%)	59 (36.2)	36 (31.9)	23 (46)	0.006
AIS extremities ≥ 3 (%)	28 (17.2)	16 (14.2)	12 (24)	0.132

*Note:* Data were presented as a number (percentage) and median IQR (25%–75%).

The most commonly associated injured organs were the liver (17.8%), ribs (17.2%), hemopneumothorax (16.6%), upper limbs (15.3%), and lower limbs (17.8%). Although TBI and pelvic fractures had a relatively lower incidence of associated injuries, TBI and pelvic fracture were significantly more frequent in the MT (+) group than in the MT (−) group (26% vs. 4.4%, *p* < 0.001 and 26% vs. 6.2%, *p* < 0.001, respectively). A comparison of the incidence of each specific injury between the two groups is presented in Table 4.

**TABLE 4:** Associated injuries of the patients with BBMI according to the massive transfusion.

	Overall (*N* = 163)	MT (−) (*n* = 113)	MT (+) (*n* = 50)	*p* value
Spleen (%)	13 (8)	8 (7.1)	5 (10)	0.540
Liver (%)	29 (17.8)	18 (15.9)	11 (22)	0.350
Pancreas (%)	9 (5.5)	5 (4.4)	4 (8)	0.458
Urinary bladder (%)	2 (1.2)	1 (0.9)	1 (2)	0.521
Kidney (%)	10 (6.1)	7 (6.2)	3 (6)	1.000
Diaphragm (%)	6 (3.7)	3 (2.7)	3 (6)	0.372
Vessel (%)	23 (14.1)	10 (8.8)	13 (26)	0.004
Traumatic brain injury (%)	18 (11)	5 (4.4)	13 (26)	< 0.001
Skull fracture (%)	4 (2.5)	2 (1.8)	2 (4)	0.587
Facial bone fracture (%)	16 (9.8)	9 (8)	7 (14)	0.259
Cervical spine fracture (%)	4 (2.5)	2 (1.8)	2 (4)	0.587
Lung contusion (%)	19 (11.7)	10 (8.8)	9 (18)	0.093
Rib fracture (%)	28 (17.2)	12 (10.6)	16 (32)	0.001
Clavicle fracture (%)	10 (6.1)	3 (2.7)	7 (14)	0.010
Scapula fracture (%)	3 (1.8)	2 (1.8)	1 (2)	1.000
Hemopneumothorax (%)	27 (16.6)	16 (14.2)	11 (22)	0.214
Thoracic spine fracture (%)	3 (1.8)	2 (1.8)	1 (2)	1.000
Lumbar spine fracture (%)	8 (4.9)	5 (4.4)	3 (6)	0.702
Pelvis fracture (%)	20 (12.3)	7 (6.2)	13 (26)	< 0.001
Upper limb fracture (%)	25 (15.3)	17 (15)	8 (16)	0.876
Lower limb fracture (%)	29 (17.8)	17 (15)	12 (24)	0.168

*Note:* Data were presented as a number (percentage).

### 3.4. Overall Morbidities

**FIGURE 2:**
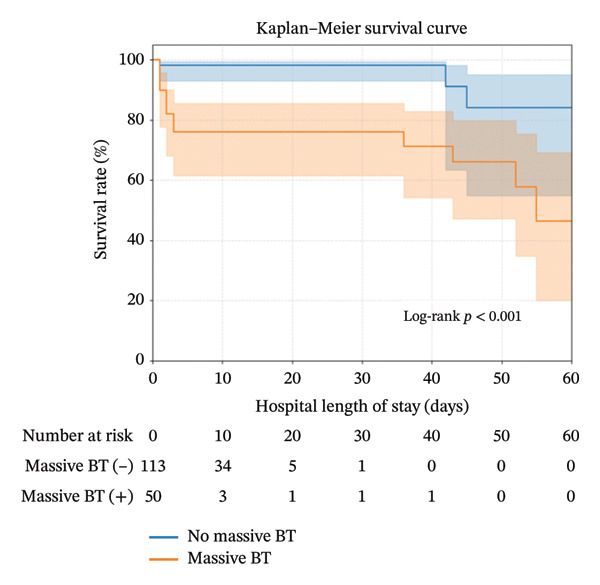
In‐hospital survival rate. Kaplan–Meier curves (95% CI) displaying in‐hospital survival for patients who experienced surgical blunt bowel mesentery injury in groups with and without massive transfusion.

The morbidity rates are shown in Table 5. There were no significant differences in the overall morbidity rates between the groups, except for unplanned ventilation (*p* < 0.001), intraabdominal abscess (*p* = 0.015), coagulopathy (*p* < 0.001), acute renal failure (*p* < 0.001), acidosis (*p* < 0.001), acute respiratory distress syndrome (*p* = 0.031), abdominal compartment syndrome (*p* = 0.004), return to operation room (*p* = 0.012), and hemodialysis intervention (*p* = 0.028), which were significantly more common in the MT(+) group than in the MT(−) group. Additionally, according to the results of the Kaplan–Meier analysis, patients in the MT‐positive group had a significantly higher and earlier rate of complications than those in the MT‐negative patients (*p* < 0.001) (Figure 2).

**TABLE 5:** Reason for morbidity comparison.

	Overall (*N* = 163)	MT (−) (*n* = 113)	MT (+) (*n* = 50)	*p* value
Sepsis (%)	25 (15.3)	16 (14.2)	9 (18)	0.530
Pneumonia (%)	25 (15.3)	15 (13.3)	10 (20)	0.272
Septic shock (%)	10 (6.1)	5 (4.4)	5 (10)	0.178
Unplanned ventilator (%)	33 (20.2)	14 (12.4)	19 (38)	< 0.001
Intra‐abdominal abscess (%)	18 (11)	8 (7.1)	10 (20)	0.015
Leakage (%)	9 (5.5)	6 (5.3)	3 (6)	1.000
Coagulopathy (%)	64 (39.3)	26 (23)	38 (76)	< 0.001
Acute renal failure (%)	60 (36.8)	31 (27.4)	29 (58)	< 0.001
Acidosis (%)	49 (30.1)	19 (16.8)	30 (60)	< 0.001
Urinary tract infection (%)	26 (16)	14 (12.4)	12 (24)	0.062
Stroke (%)	4 (2.5)	2 (1.8)	2 (4)	0.587
Pulmonary embolism (%)	2 (1.2)	2 (1.8)	0 (0)	1.000
ARDS (%)	5 (3.1)	1 (0.9)	4 (8)	0.031
Pleural effusion (%)	26 (16)	14 (12.4)	12 (24)	0.062
Enterocutaneous fistula (%)	2 (1.2)	1 (0.9)	1 (2)	0.521
Wound infection (%)	33 (20.2)	20 (17.7)	13 (26)	0.224
Wound dehiscence (%)	8 (4.9)	4 (3.5)	4 (8)	0.251
Abdomen compartment (%)	7 (4.3)	1 (0.9)	6 (12)	0.004
Tracheostomy (%)	3 (1.8)	1 (0.9)	2 (4)	0.223
ECMO (%)	4 (2.5)	1 (0.9)	3 (6)	0.086
Return to OR (%)	25 (15.3)	12 (10.6)	13 (26)	0.012
Hemodialysis (%)	3 (1.8)	0 (0)	3 (6)	0.028
Intestinal obstruction (%)	3 (1.8)	2 (1.8)	1 (2)	1.000

*Note:* Data were presented as a number (percentage).

Abbreviations: ARDS, acute respiratory distress syndrome; ECMO, extracorporeal membrane oxygenation; OR, operation room.

## 4. Discussion

**FIGURE 3:**
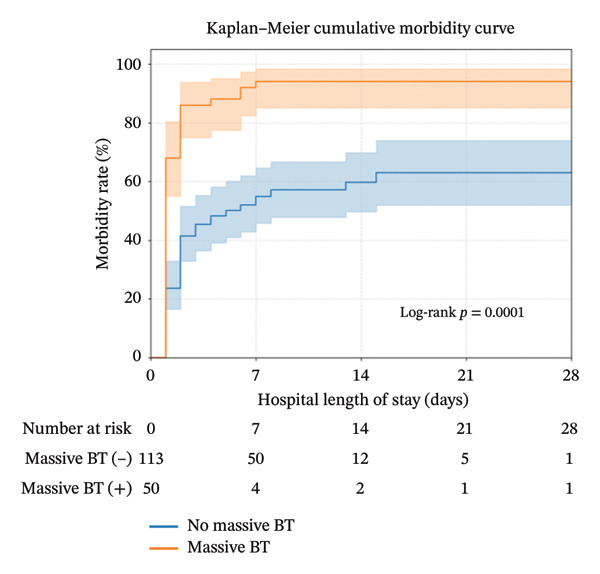
Kaplan–Meier curves (95% CI) displaying in‐hospital morbidity for patients who experienced surgical blunt bowel mesentery injury in groups with and without massive transfusion.

The principal treatment for BBMI is based on early diagnosis, timely operation, and attempted resuscitation. However, apart from early therapeutic laparotomy, timely recognition of patients in need of rapid activation using the MTP could be another challenge before laparotomy. Although the need for MT to resuscitate patients with BBMI is well known, fewer literature studies are associated with this issue. Niederee et al. conducted a 11‐year retrospective study associated with outcome of delayed diagnosis (> 24 h) in patients with BBMI and reported that the patients with diagnosis within 24 h had received mean 8.5 unites blood products and 26.7% mortality rate, whereas patients with diagnosis beyond 24 h had received 11.7 units blood products and 36.4% mortality rate [7]. Our results show that there are approximately 31% patients in need of MT after BBMI, who present 32% overall mortality rate and 8% mortality rate within 24 h, respectively. The median administered PRBCs within 24 h in the MT(+) and MT(−) group were 16 and 2 U, respectively. The more blood products administered in the MT (+) group may result from the higher injury severity with ISS ≥ 16 of 80% and ISS ≥ 25 of 58%, respectively, or involvement of intrinsic mesentery injury. It may also result from the higher incidence of presence with shock episode (94%) before laparotomy in the MT(+) group, which lead to the need for active resuscitation with more blood volume. These abovementioned presentations of BBMI can reflect its characteristics of multitrauma, difficult management, and high mortality rate. Accordingly, it is helpful to explore the relationship of MT in multitraumatized patients with concomitant BBMI (Figure 3).

To predict the MT for patients after abdominal trauma, El‐Menyar et al. created FASILA scores using the acronym FAST, SI, and initial serum lactate to investigate the implications of MTP activation, exploratory laparotomy (ExpLap), and mortality and proposed that from FASILA scores of 4–6 points, the percentage of ExpLap increases from 28% to 85%, whereas the MTP activation increases from 17% to 68% [4]. They validated the novel FASILA score as superior to the RABT, SI, and ABC scores in the prediction of MTP and BT using receiver operating characteristic analysis. Although the study included all injured organs after abdominal trauma, either penetration or blunt mechanisms, it seemed impractical in the prediction of ExLap, as there were fewer BAT patients who still required ExLap in the era of NOM [6]. El‐Menyar et al. [9] also conducted another retrospective analysis with abdominal trauma patients sustaining solid organ injury, using SI ≥ 0.8 to predict the transfusion, and concluded that on multivariate analysis, although SI had significant prediction of transfusion and MTP activation, it failed to show significant prediction of ExLap. Therefore, we thought that it would be interesting to explore the association between MT and BAT in a different prediction model in terms of the investigation of associated injury of hollow organ injury.

Our study shows that besides the involvement of mesenteric injury, TBI and pelvic fracture are significant risk factors for the prediction of MT in patients with surgical BBMI after multivariate analysis. Although the BMI was significant in the univariate analysis, it no longer continued the significance in the multivariate analysis after controlling other confounders. We thought that it may be due to the BMI was not independently associated with the ISS, morbidity, or mortality after trauma, which existed a J‐shaped relationship between the trauma and clinical results [13].

As expected, pelvic fracture was identified as a significant risk factor. However, it is noteworthy that TBI remained a significant risk factor even after controlling for other confounding variables. Similar to BAT, TBI is associated with substantial morbidity and mortality following trauma; nevertheless, intracerebral hemorrhage (ICH) does not theoretically result in significant blood loss. After reviewing the variables included in the abovementioned predictive scores for MT, it is difficult to establish a direct implication between MT and TBI. This association may instead be mediated by TBI‐associated coagulopathy, which contributes to bleeding progression and may increase transfusion requirements [14]. A meta‐analysis has reported that coagulopathy occurs in approximately 35% of patients with isolated TBI [15]. Peiniger et al. reported that patients sustaining major trauma (ISS ≥ 16) with concomitant TBI (AIS ≥ 3) who received a MTP with a high FFP:PRBC ratio (> 1:2) demonstrated improved survival [16]. This finding supports the concept of early coagulopathy in severely injured patients with TBI. In addition, the observed association may be influenced by MTP‐related balanced transfusion strategies, which may variably affect outcomes in patients with TBI and MT across different institutions. Nevertheless, causation remains complex and is likely influenced by multiple factors, including hemorrhagic shock, anemia, and associated systemic injuries. Overall, evidence specifically addressing MT in patients with TBI remains limited, underscoring the need for larger prospective studies focusing on MT in patients with TBI combined with visceral organ injury following BAT.

Our results showed that pelvic fracture is another significant predictor of MT in patients sustaining BBMI with concomitant pelvic fracture, which is similar to other scoring systems that use pelvic fracture as a variable, such as the TASH, PWH, and RABT scores [2, 5]. Patients with BAT who sustain pelvic fractures with concomitant intraperitoneal and retroperitoneal hemorrhage were thought to have a high possibility of requiring laparotomy and activation of the MTP. It was found that patients receiving laparotomy needed a mean BT of 914.5 mL, patients receiving laparotomy plus subsequent TAE required a mean BT of 1542.8 mL [17]. Additionally, patients with concomitant unstable hemodynamic and pelvic fractures were difficult to manage, in whom intra‐abdominal injury was easily overlooked, particularly BBMI, with an 86% missed rate. These difficult‐to‐manage patients would require initial TAE for hemostasis and post‐TAE laparotomy for intra‐abdominal missed injuries, requiring a mean transfusion of 2016.7 mL [10]. If the intra‐abdominal lesion requiring operation is not recognized in time in such patients and the required laparotomy is delayed by different times, they would require a mean transfusion of 1035.7 mL with a delay < 6 h, 1625.0 mL with a delay of 6–24 h, and 3291.7 mL with a delay > 24 h [10]. It is well known that hemodynamic instability frequently occurs in patients with complex pelvic fractures, although in our present series, only 12.3% (20/163) of patients had combined pelvic fractures without stratifying stable or unstable fracture patterns, which had insufficient power to corroborate it as a predictor. Previous evidence had reported that the pattern, stability, and severity of pelvic fractures do not play significant roles in predicting mortality in patients with blunt pelvic fractures, because even a low‐energy pelvic fracture would lead to a significant life threatening [18, 19]. In prior scores, PWH and TASH used displaced or unstable pelvic fractures as variables to predict MT [2], which would decrease the sensitivity of missing the opportunity to activate the MTP. On the other hand, a recent RABT score using any pelvic fracture as a variable was validated in Canadian Level I centers, and it was reported that after subanalysis for patients with only unstable pelvic fractures compared with any pelvic fractures, the RABT score had decreased sensitivity from 62% to 55% but improved specificity from 63% to 68% without a change in the positive predictive value (23%) and negative predictive value (90%) [3]. Accordingly, the patients following any pelvic fracture should have a hemoglobin assessment and alert the clinicians to the possibility of transfusion. Based on our findings, we suggested if the trauma surgeon faced these patients with the presence of unstable hemodynamic status or higher injury severity, or significant hemoperitoneum [20] and combination of pelvic fracture and high suspicion of BBMI from CT image, they should get involved actively for these patients.

In our study, we did not include the ISS in the multivariate logistic regression because the ISS interacts with other associated injuries. The ISS is an anatomical scoring system that can provide a good severity assessment; however, it can only select an injured organ within a body region and fails to predict prognosis. The MT(+) group had a higher ISS and BT amounts and percentages, which could explain the early presence of morbidity and mortality in the MT(+) group, regardless of its inherently higher injury severity or complications of receiving MT [1]. Furthermore, a higher ISS resulted from a higher incidence of ICH in the MT(+) group. Although ICH can contribute to a high ISS, ICH usually does not require transfusion, which is another reason we did not include the ISS in the logistic regression. Our results showed that TBI and pelvic fractures were risk factors for MT in multitraumatized patients with concomitant BBMI. Additionally, the association between TBI and hemorrhagic pelvic fractures has not been discussed in prior studies. Kido et al. [18] conducted a retrospective analysis including 102 patients with concomitant fatal bleeding pelvic fracture and severe associated injuries (AIS ≥ 3) and concluded that TBI and shock were significant independent predictors in mortality within 24 h, indicating that TBI had a higher risk of mortality than other body parts injury in patients with fatal bleeding pelvic fracture, whose pelvic artery active bleeding was identified by enhanced CT. In other words, this finding seemed to reflect that although TBI had a worse outcome inherently in trauma, as expected, it cannot exclude the fact that TBI may advance to hemorrhagic shock in the acute stage in patients with fatally bleeding pelvic fractures. However, the authors did not mention the relationship between BTs in their study. Accordingly, future studies on TBI and bleeding after BAT are warranted.

### 4.1. Limitations

There are some limitations in our study. First, its retrospective design with 14‐year duration including differing approaches and interpretations of image studies, institutional experience, and small sample size may introduce occult biases and weaknesses in terms of powerful statistical significance and generalization. These factors may also contribute to wide CIs and potentially inflated or unstable odds ratio estimates. Secondly, our study mainly focused on MT in patients, however, we did not have data on the quantity of hemoperitoneum on CT or FAST [18], given that some patients was transferred from other hospitals at holidays so as to miss to reserve the copy of images in the PACS system. Otherwise, the most variables including our data can be found in the trauma register or electronic medical record. Third, we did not stratify the severity of every concomitant or associated injury, which introduces bias in the consistency of severity. Fourth, temporal changes in trauma management strategies and transfusion practices thought out the extended study period, particularly evolving transfusion thresholds and resuscitation guidelines, may have affected patient outcome and limited the direct applicability of our findings to trauma centers with different contemporary practices. Although our hospital’s MTP was established in 2016 based on a 1:1 RBC:FFP ratio, its practical clinical implementation was not realized until 2018, coinciding with the procurement of rapid blood warmers. Consequently, within the 14‐year study period, the number of cases activating the MTP in this study was small; therefore, the impact of MTP activation on this study is limited. Despite these weaknesses, our study provided important insights into the predictions of MT in BBMI and showed that the associated injury with TBI or pelvic fracture had a significant impact on multitraumatized patients with BBMI requiring MT. We hoped that we could innovatively seek easy, nonlaboratory, and rapid assessment models to predict the requirement of MT during the expeditious diagnosis and resuscitation course for the purpose of dealing with inherent trauma nature with a dynamic change and aid in decision‐making for treating difficult‐to‐manage patients with surgical BBMI.

## 5. Conclusions

Although BBMI is a rare entity in the ED, almost half of the patients (45%) exhibited shock episodes before laparotomy, one‐third of patients required MT in the acute phase, and one‐third of mortality in those need MT, which indicates the critical status regarding managing multitraumatized patients with surgical BBMI. We found that pelvic fracture and TBI were statistically significant risk factors for an associated injury in multitraumatized patients with surgical BBMI requiring MT. When BBMI patients are assessed in the ED with the presence of hemodynamic instability, potential bleeding according to existing scoring models in trauma, and higher injury severity with concomitant of TBI or pelvic fracture, these patients may expect the activation of MTP without therapeutic delay.

**Nomenclature**
ABC: assessment of blood consumption
AIS: abbreviated injury score
ARDS: acute respiratory distress syndrome
BAT: blunt abdominal trauma
BBMI: blunt bowel mesenteric injuries
BMI: body mass index
BT: blood transfusion
CT: computed tomography
ECMO: extracorporeal membrane oxygenation
ED: emergency department
ExpLap: exploratory laparotomy
FAST: focused assessment with sonography in trauma
FFP: fresh frozen plasma
GCS: Glasgow Coma Scale
HR: heart rate
ICH: intracerebral hemorrhage
ICP: intracranial hypertension
ICU: intensive care unit
IRB: Institutional Review Board
ISS: injury severity score
LOS: length of stay
MT: massive transfusion
MTP: massive transfusion protocol
NISS: new injury severity score
NOM: nonoperative management
OR: odds ratio
PWH: Prince of Wales Hospital
RABT: Revised Assessment of Bleeding and Transfusion
RBC: red blood cell
RR: respiratory rate
RTS: revised trauma score
SBP: systolic blood pressure
SI: shock index
SIRS: systemic inflammatory response syndrome
TASH: trauma‐associated severe hemorrhage
TBI: traumatic brain injury
TRISS: trauma resuscitation injury severity score

## Author Contributions

Conceptualization, Ting‐Min Hsieh and Fu‐Jen Cheng; formal analysis, Po‐Chun Chuang; writing, Chun‐Ting Liu; data curation, Bei‐Yu Wu; and writing–original draft preparation, Fu‐Jen Cheng and Ching‐Hua Hsieh.

## Funding

This research received no external funding.

## Disclosure

All authors have read and agreed to the published version of the manuscript. This manuscript was previously made available as a preprint on Research Square [21].

## Ethics Statement

This study was approved by the Institutional Review Board of Chang Gung Memorial Hospital (approval number: 202500868B0) and performed in accordance with the ethical guidelines of the 1964 Declaration of Helsinki and its later amendments or comparable ethical standards. The need for obtaining informed consent was waived according to IRB regulations.

## Consent

Please see the ethics statement.

## Conflicts of Interest

The authors declare no conflicts of interest.

## Supporting Information

The following supporting information are available:

Table S1: Correlation matrix and VIFs assessing multicollinearity among candidate predictors in the regression model for massive transfusion.

Table S2: Predictors for massive transfusion after excluding early deaths (death within 24 h).

Table S3: Predictors of massive transfusion stratified by the study period (2009–2015 vs. 2016–2024).

Table S4: Reduced multivariate logistic regression model for predictors of massive transfusion in patients with blunt bowel and mesenteric injury (BBMI).

Table S5: Predictors of massive transfusion using Firth penalized logistic regression.

## Supporting information


**Supporting Information** Additional supporting information can be found online in the Supporting Information section.

## Data Availability

Data were obtained from Chang Gung Research Database and are available by corresponding with the author and obtaining permission.
