# Supplementary material for: Predictors of Massive Transfusion in Patients With Hollow Organ Injury After Blunt Multiple Trauma: A Cohort of Blunt Bowel Mesenteric Injury
Source: Emerg Med Int. 2026 Apr 13;2026:5286788. doi: 10.1155/emmi/5286788 (PMC13071859; doi:10.1155/emmi/5286788)
Supplement: Supplementary file 1 — Supporting Information Additional supporting information can be found online in the Supporting Information section. [file EMMI-2026-5286788-s001.docx]

Supplementary Table S1. Correlation matrix and variance inflation factors (VIF) assessing multicollinearity among candidate predictors in the regression model for massive transfusion.

|  | **VIF** |
| --- | --- |
| **Age** | 1.237 |
| **Male sex** | 1.128 |
| **Body mass index** | 1.282 |
| **Vessel** | 1.253 |
| **Traumatic brain injury** | 1.290 |
| **Lung contusion** | 1.493 |
| **Rib fracture** | 1.807 |
| **Hemopneumothorax** | 1.689 |
| **Pelvic fracture** | 1.246 |
| **Low limb fracture** | 1.265 |
| **Isolated colon injury** | 1.426 |
| **Isolated mesentery injury** | 2.116 |
| **combined injury** | 2.032 |

Supplementary Table S2 Predictors for massive transfusion excluding early deaths (death within 24 hours)

|  | **AOR (95% CI)** | ***p* value** |
| --- | --- | --- |
| **Age** | 0.99 (0.97-1.02) | 0.618 |
| **Male sex** | 1.87 (0.55-6.36) | 0.316 |
| **Body mass index** | 1.06 (0.95-1.18) | 0.324 |
| **Vessel** | 1.77 (0.57-5.52) | 0.327 |
| **Traumatic brain injury** | 6.59 (1.56-27.89) | 0.010 |
| **Lung contusion** | 0.79 (0.19-3.32) | 0.749 |
| **Rib fracture** | 1.68 (0.39-7.25) | 0.489 |
| **Hemopneumothorax** | 0.9 (0.17-4.83) | 0.901 |
| **Pelvic fracture** | 6.09 (1.48-25.03) | 0.012 |
| **Low limb fracture** | 1.29 (0.4-4.16) | 0.668 |
| **Isolated colon injury** | 0.69 (0.03-16.39) | 0.817 |
| **Isolated mesentery injury** | 14.9 (1.54-145.16) | 0.020 |
| **combined injury** | 22.75 (2.38-217.52) | 0.007 |

Supplementary Table S3. Predictors of massive transfusion stratified by study period

|  | **2009-2015 (n=58)** | | **2016-2024 (n=105)** | |
| --- | --- | --- | --- | --- |
|  | **AOR (95% CI)** | ***p* value** | **AOR (95% CI)** | ***p* value** |
| **Age** | 0.99 (0.95–1.04) | 0.802 | 0.99 (0.96–1.03) | 0.674 |
| **Male sex** | 0.79 (0.06–10.37) | 0.855 | 2.27 (0.43–12.18) | 0.337 |
| **Body mass index** | 0.96 (0.72–1.27) | 0.75 | 1.13 (0.97–1.31) | 0.12 |
| **Vessel** | 0.44 (0.04–5.62) | 0.531 | 2.88 (0.65–12.75) | 0.164 |
| **Traumatic brain injury** | 12.06 (0.59–245.24) | 0.105 | 10.69 (1.44–79.55) | 0.021 |
| **Lung contusion** | 4.06 (0.00–8339.74) | 0.719 | 0.85 (0.14–5.27) | 0.859 |
| **Rib fracture** | 15.95 (0.42–606.89) | 0.136 | 0.61 (0.09–4.27) | 0.62 |
| **Hemopneumothorax** | 0.05 (0.00–2.22) | 0.122 | 3.94 (0.27–57.01) | 0.315 |
| **Pelvic fracture** | 17.12 (1.06–276.09) | 0.045 | 7.89 (0.95–65.25) | 0.055 |
| **Low limb fracture** | 1.21 (0.15–9.74) | 0.861 | 1.21 (0.22–6.55) | 0.823 |
| **Isolated colon injury** | 0.00 (0.00–NA) | 0.999 | NA | 0.998 |
| **Isolated mesentery injury** | 4.98 (0.29–86.71) | 0.271 | NA | 0.998 |
| **combined injury** | 0.99 (0.95–1.04) | 0.802 | NA | 0.998 |

Supplementary Table S4 Reduced multivariate logistic regression model for predictors of massive transfusion in patients with blunt bowel and mesenteric injury (BBMI)

|  | **AOR (95% CI)** | **p value** |
| --- | --- | --- |
| Age | 0.99 (0.97–1.02) | 0.564 |
| Male sex | 1.37 (0.52–3.60) | 0.522 |
| **Traumatic brain injury** | **7.22 (2.28–22.90)** | **0.001** |
| **Pelvic fracture** | **4.88 (1.69–14.04)** | **0.003** |

Supplementary Table S5. Predictors of massive transfusion using Firth penalized logistic regression.

|  | **AOR** | **95% CI** | |
| --- | --- | --- | --- |
| **Age** | 0.995 | 0.970 | 1.020 |
| **Male sex** | 1.587 | 0.550 | 5.022 |
| **Body mass index** | 1.047 | 0.945 | 1.164 |
| **Vessel** | 1.398 | 0.499 | 3.953 |
| **Traumatic brain injury** | 5.379 | 1.579 | 21.371 |
| **Lung contusion** | 0.852 | 0.218 | 3.129 |
| **Rib fracture** | 1.950 | 0.536 | 7.638 |
| **Hemopneumothorax** | 0.770 | 0.155 | 3.449 |
| **Pelvic fracture** | 4.675 | 1.370 | 19.068 |
| **Low limb fracture** | 1.101 | 0.366 | 3.231 |
| **Isolated colon injury** | 0.916 | 0.054 | 14.109 |
| **Isolated mesentery injury** | 12.141 | 2.405 | 124.719 |
| **combined injury** | 14.018 | 2.832 | 144.396 |
